# From central to pulmonary capillary blood volume: Evidence for a non‐linear relationship with pulmonary diffusing capacity

**DOI:** 10.1113/JP292195

**Published:** 2026-08-23

**Authors:** Ronan M. G. Berg

**Affiliations:** ^1^ Centre for Physical Activity Research Copenhagen University Hospital – Rigshospitalet Copenhagen Denmark; ^2^ Department of Clinical Physiology and Nuclear Medicine Copenhagen University Hospital – Rigshospitalet Copenhagen Denmark; ^3^ Department of Clinical Medicine, Faculty of Health and Medical Sciences University of Copenhagen Copenhagen Denmark; ^4^ Neurovascular Research Laboratory, Faculty of Life Sciences and Education University of South Wales Pontypridd UK

**Keywords:** central blood volume, diffusing capacity, gas exchange

Central blood volume (CBV) is defined as the blood volume contained within the pulmonary vasculature, the cardiac chambers and the major intrathoracic extrapulmonary blood vessels (Gauer & Henry, 1963). It changes acutely in response to various physiological stressors, including postural changes, hypo‐ and hypergravity and acute exercise, among others, through effects on the cardiopulmonary baroreceptor system, an important component of the autonomic regulation of cardiovascular function. Furthermore, in the healthy resting state approximately 5% to 7% of CBV is located at the pulmonary capillary level. Although pulmonary capillaries appear to be continuously perfused with plasma at rest, at least in the healthy state, only approximately half are concurrently perfused with red blood cells (Wagner et al., 1999). The remaining capillaries can be recruited and distended, and perhaps also de‐recruited and de‐distended, in response to changes in cardiac output and pulmonary vascular pressures, with concomitant changes in CBV (Hsia et al., 2016).

Hitherto, the extent to which changes in CBV lead to changes in pulmonary capillary blood volume, and how such changes ultimately affect the diffusive conductance for pulmonary gas transfer, that is, the pulmonary diffusing capacity, has remained unknown. That changes with the study by D'Souza et al., published in this issue of *The Journal of Physiology* (D'Souza et al., 2026). In this study the authors manipulated CBV by exposing healthy participants to graded lower‐body positive pressure (LBPP) and lower‐body negative pressure (LBNP) to increase and decrease CBV, respectively. Changes in CBV were monitored using impedance cardiography‐derived thoracic fluid content as a surrogate measure, whereas pulmonary diffusing capacity for carbon monoxide and nitric oxide was determined at each pressure level. Owing to the different specific blood conductances of these test gases, arising primarily from their different haemoglobin reaction kinetics, this approach permitted partitioning of pulmonary diffusing capacity into alveolar‐capillary membrane conductance and pulmonary capillary blood volume using the Roughton–Forster equation (Munkholm et al., 2018). Measurements were obtained across the full range of experimental conditions from LBNP at −60 mmHg to LBPP at +30 mmHg.

As expected, LBNP‐ and LBPP‐induced changes in CBV were accompanied by corresponding changes in pulmonary capillary blood volume, consistent with pulmonary capillary recruitment and distension during increases in CBV and vice versa during reductions in CBV. Curiously, these changes translated into alterations in pulmonary diffusing capacity only during reductions in CBV, because alveolar‐capillary membrane conductance exhibited a seemingly paradoxical decrease during LBPP. Rather than attributing these observations to an isolated effect of increased CBV, D'Souza et al. argue that the findings are better explained by the combined effects of a reduction in alveolar volume during LBPP together with blood volume‐dependent changes in alveolar‐capillary geometry (D'Souza et al., 2026). Regardless of the underlying mechanism the findings demonstrate that, despite parallel changes in pulmonary capillary blood volume, the integrated pulmonary diffusing capacity response to decreases and increases in CBV is non‐linear in humans.

As highlighted by D'Souza et al., their study identifies pulmonary capillary blood volume as a key intermediary linking changes in CBV to pulmonary gas exchange (D'Souza et al., 2026). Beyond addressing a fundamental question in integrative physiology, these findings also have broader clinical and applied implications. They may advance our understanding of how altered CBV influences pulmonary gas exchange in conditions such as severe haemorrhage, postural tachycardia syndrome, acute heart failure, chronic obstructive pulmonary disease (COPD) and cirrhosis, while also being relevant to understanding physiological responses to environmental and operational stressors that alter CBV, including those encountered in aviation, spaceflight and diving medicine. Whether the non‐linear relationship to pulmonary diffusing capacity identified by D'Souza et al. is specific to acute lower‐body pressure‐induced changes in CBV, or whether it represents a more general principle of pulmonary physiology that extends to other perturbations causing acute or chronic alterations in CBV, remains an open question.

## Additional information

### Competing interests

No competing interests declared.

### Author contributions

R.M.G.B.: conception, data interpretation, first draft and revisions. The author approved the final version of the manuscript and agrees to be accountable for all aspects of the work in ensuring that questions related to the accuracy or integrity of any part of the work are appropriately investigated and resolved.

### Funding

The Centre for Physical Activity Research (CFAS) is supported by TrygFonden (grants IDs: 101390, 20045, 125132 and 177225). The funders had no role in study design, data collection and analysis, decision to publish or preparation of the manuscript.

### Disclosures

None.

### Generative AI statement

During the preparation of this manuscript ChatGPT (OpenAI, GPT‐5.5) was used solely for spelling and grammatical editing. No generative artificial intelligence was used for the conceptualisation of the study, study design, data analysis, interpretation of the findings or the generation of substantive scientific content. The author reviewed and approved all revisions and takes full responsibility for the content of the manuscript.

## Supporting information


Peer Review History
