# Supplementary material for: From central to pulmonary capillary blood volume: Evidence for a non‐linear relationship with pulmonary diffusing capacity
Source: J Physiol. 2026 Aug 23;604(18):7523–4. doi: 10.1113/JP292195 (PMC13577676; doi:10.1113/JP292195)
Supplement: Supplementary file 1 [file TJP-604-7523-s001.pdf]

The Journal of Physiology

From central to pulmonary capillary blood volume: Evidence for a nonlinear relationship with pulmonary diffusing capacity

Ronan M. G. Berg
DOI: 10.1113/JP292195

Corresponding author(s): Ronan Berg (ronan@sund.ku.dk)

The following individual(s) involved in review of this submission have agreed to reveal their identity: Michael K Stickland (Referee #1)

Review Timeline:

Submission Date:	26-Jul-2026
Accepted:	31-Jul-2026

Senior Editor: Bjorn Knollmann

Reviewing Editor: Joseph Welch

Transaction Report:

(Note: With the exception of the correction of typographical or spelling errors that could be a source of ambiguity, letters and reports are not edited. Depending on transfer agreements, referee reports obtained elsewhere may or may not be included in this compilation. Referee reports are anonymous unless the Referee chooses to sign their reports.)

Dear Professor Berg,

Re: JP-P-2026-292195 "**From central to pulmonary capillary blood volume: Evidence for a nonlinear relationship with pulmonary diffusing capacity**" by Ronan M. G. Berg

We are pleased to tell you that your paper has been accepted for publication in The Journal of Physiology.

The last Word (or similar) version of the manuscript provided will be used by the Production Editor to prepare your proof. When this is ready you will receive an email containing a link to Wiley's Online Proofing System. The proof should be thoroughly checked and corrected as promptly as possible.

Authors should note that it is too late at this point to offer corrections prior to proofreading. Major corrections at proof stage, such as changes to figures, will be referred to the Editors for approval before they can be incorporated. Only minor changes, such as to style and consistency, should be made at proof stage. Changes that need to be made after proof stage will usually require a formal correction notice.
All queries at proof stage should be sent to: TJP@wiley.com.

Following copyediting and typesetting the accepted version will be published online.

Please note that Perspective articles are not typically covered by institutional open access agreements with our publisher, Wiley. Wiley do not offer article processing charge (APC) discounts for smaller article types in hybrid subscription journals, meaning that if you wish for your Perspective to be published Open Access, you will have to pay the full APC. As such, we recommend authors publish Perspectives 'behind the paywall', where they will become freely accessible after a 12-month embargo (i.e. please select the NON open access option via 'Wiley Authors' during proofing).

Should you wish to pay for Open Access, you will be able to place an order by logging into Wiley Author services.

• You can check if your funder or institution has a Wiley Open Access Account here:
https://authors.wiley.com/author-resources/Journal-Authors/open-access/author-compliance-tool.html

Yours sincerely,

Bjorn Knollmann
Senior Editor
The Journal of Physiology

IMPORTANT POINTS TO NOTE FOLLOWING ACCEPTANCE OF YOUR PAPER:

• IMPORTANT NOTICE ABOUT OPEN ACCESS: To assist authors whose funding agencies mandate immediate public access to published research findings, The Journal of Physiology allows authors to pay an Open Access (OA) fee to have their papers made freely available immediately on publication.

The Corresponding Author will receive an email from Wiley with details on how to register or log in to Wiley Authors where you will be able to place an order.

• You can check if your funder or institution has a Wiley Open Access Account here: https://authors.wiley.com/author-resources/Journal-Authors/open-access/author-compliance-tool.html

MEMBER DISCOUNT: Corresponding authors who are Members of the Physiological Society are eligible for a 20% discount on the cost of the Article Processing Charge (APC). To claim this discount, the Corresponding Author will need to log in to the Physiological Society Membership portal and retrieve the discount code (https://www.physoc.org/journals-and-media/journals/journal-discount-for-members/). This code should then be quoted in the billing section of Wiley Authors when selecting the open access option.

• TRANSPARENT PEER REVIEW POLICY: To improve the transparency of its peer review process, The Journal of Physiology publishes online as supporting information the peer review history of all articles accepted for publication. Readers will have access to decision letters, including Editors' comments and referee reports, for each version of the manuscript as well as any author responses to peer review comments. Referees can decide whether or not they wish to be named on the peer review history document.

• You can help your research get the attention it deserves! Check out Wiley's free Promotion Guide for best-practice recommendations for promoting your work at: www.wileyauthors.com/eeo/guide. You can learn more about Wiley Editing

Services which offers professional video, design, and writing services to create shareable video abstracts, infographics, conference posters, lay summaries, and research news stories for your research at: www.wileyauthors.com/eeo/promotion.

• If you would like to receive our 'Research Roundup', a monthly newsletter highlighting the cutting-edge research published in The Physiological Society's family of journals (The Journal of Physiology, Experimental Physiology, Physiological Reports, The Journal of Nutritional Physiology and The Journal of Precision Medicine: Health and Disease), please click this link, fill in your name and email address and select 'Research Roundup':
https://www.physoc.org/journals-and-media/membernews

EDITOR COMMENTS

Reviewing Editor:

Thank you for submitting your Perspectives article to The Journal of Physiology. The article is an excellent summary of the work of D'Souza et al. The reviewer confirms that the article is factually correct. I also have no further comments. Thank you for your contribution to The Journal of Physiology.

Senior Editor:

The Perspective is acceptable for publication.

REFEREE COMMENTS

Referee #1:

We thank Dr Berg for his interest in our paper and his insightful commentary which we very much enjoyed reading.

The commentary is factually correct, and we have no further comments or critique. Thank you.
